# Lymph Nodes Draining Infections Investigated by PET and Immunohistochemistry in a Juvenile Porcine Model

**DOI:** 10.3390/molecules27092792

**Published:** 2022-04-27

**Authors:** Pia Afzelius, Malene Kjelin Morsing, Ole Lerberg Nielsen, Aage Kristian Olsen Alstrup, Svend Borup Jensen, Lars Jødal

**Affiliations:** 1 Department of Clinical Physiology and Nuclear Medicine Zealand Koege, University Hospital of Copenhagen, 4600 Copenhagen, Denmark; 2 Department of Nuclear Medicine, Aalborg University Hospital, 9000 Aalborg, Denmark; svbj@rn.dk (S.B.J.); lajo@rn.dk (L.J.); 3 Department of Veterinary and Animal Sciences, University of Copenhagen, 1870 Frederiksberg C, Denmark; malenekjelin@gmail.com (M.K.M.); olelerbergnielsen@gmail.com (O.L.N.); 4 Department of Nuclear Medicine and PET, Aarhus University Hospital, Skejby, 8200 Aarhus, Denmark; aagols@rm.dk; 5 Department of Clinical Medicine, Aarhus University, Skejby, 8200 Aarhus, Denmark; 6 Department of Chemistry and Biosciences, Aalborg University, 9220 Aalborg, Denmark

**Keywords:** PET, [^18^F]FDG, [^11^C]methionine, lymph nodes, pig model, *Staphylococcus aureus*, Ki-67, calcium-binding leukocyte L1, IL-8, immunohistochemistry

## Abstract

Background: [^18^F]FDG and [^11^C]methionine accumulate in lymph nodes draining *S. aureus* -infected foci. The lymph nodes were characterized by weight, [^11^C]methionine- and [^18^F]FDG-positron emissions tomography (PET)/computed tomography (CT), and immunohistochemical (IHC)-staining. Methods: 20 pigs inoculated with *S. aureus* into the right femoral artery were PET/CT-scanned with [^18^F]FDG, and nine of the pigs were additionally scanned with [^11^C]methionine. Mammary, medial iliac, and popliteal lymph nodes from the left and right hind limbs were weighed. IHC-staining for calculations of area fractions of Ki-67, L1, and IL-8 positive cells was done in mammary and popliteal lymph nodes from the nine pigs. Results: The pigs developed one to six osteomyelitis foci. Some pigs developed contiguous infections of peri-osseous tissue and inoculation-site abscesses. Weights of mammary and medial iliac lymph nodes and their [^18^F]FDG maximum Standardized Uptake Values (SUV_FDGmax_) showed a significant increase in the inoculated limb compared to the left limb. Popliteal lymph node weight and their FDG uptake did not differ significantly between hind limbs. Area fractions of Ki-67 and IL-8 in the right mammary lymph nodes and SUV_Metmax_ in the right popliteal lymph nodes were significantly increased compared with the left side. Conclusion: The PET-tracers [^18^F]FDG and [^11^C]methionine, and the IHC- markers Ki-67 and IL-8, but not L1, showed increased values in lymph nodes draining soft tissues infected with *S. aureus*. The increase in [^11^C]methionine may indicate a more acute lymph node response, whereas an increase in [^18^F]FDG may indicate a more chronic response.

## 1. Introduction

### 1.1. Background and Previous Studies

Positron emission tomography (PET) is a high-resolution functional scanning modality used to disclose cells and tissues with either metabolic changes or the expression of specific ligands. The glucose analog 2-[^18^F]fluoro-2-deoxyglucose ([^18^F]FDG) is used extensively in PET for cancer diagnostics and staging, but also, to some extent, in inflammatory and infectious diseases [1]. Thus, [^18^F]FDG PET cannot distinguish malignancy from inflammation or infection [2]. Another issue is the physiological accumulation of [^18^F]FDG in normal tissues such as growth zones of juvenile bones, the heart muscle, and the brain. For a lesion in organs with high physiological uptake, the lesion-to-background uptake ratio will be correspondingly lower, making the interpretation of pathological uptake more difficult.

Amino acid tracers may be better-suited radiopharmaceuticals due to their lower physiological uptake [3,4]. They may improve the interpretation of scans by a better distinction between pathology and physiological uptake. Uptake studies have shown that methionine accumulates in inflammatory cells, and stimulation/activation of these cells increases methionine uptake [2]. Furthermore, imaging of methionine uptake may detect the early phases of tissue healing, infiltration of inflammatory cells, the formation of granulation tissue, and remodeling of tissues [3].

The overall aim of our study was to find better radiotracers for osteomyelitis (OM) in humans, and to do so, we tested several radiotracers and compared them with the golden standard for OM-detection: biopsy. However, there are no golden standards for testing radiotracers for OM. We, therefore, developed an OM model for juvenile pigs, and, to us, it makes sense to evaluate the pros and cons for the model.

Understanding the nature of the in vivo accumulation of tracers such as [^11^C]methionine (L-[*methyl*-^11^C]methionine) is crucial for interpreting PET images. Studies that identify cells, and, ideally, cell function responsible for accumulating tracers could thus provide information that allows separation of malignancy from inflammation and even separation of different stages of inflammation, and thereby guide treatment and planning of follow-up.

Normally, the staphylococci do not cause disease (asymptomatic colonization/carrier of staphylococci), but Staphylococcus aureus (*S. aureus*) can cause infections. Most common are inflammations in tears, wounds, and other lesions of the skin; childhood ulcers; and abscesses. In debilitated individuals, *S. aureus* also causes deep infections, such as osteomyelitis, osteoarthritis, arthritis, pneumonia, sepsis, and heart valve inflammation. Many procedures and, specifically, procedures performed in hospitals, such as the placement of catheters in blood vessels, drains, hip- and knee prosthesis, and surgeries, increase the risk of staphylococcal infections.

We, therefore, have tested multiple radiotracers, and improved a juvenile pig model for osteomyelitis, using *S. aureus* as the infectant due to this pathogen’s commonness in osteomyelitis [5]. Though [^18^F]FDG was the most sensitive tracer to detect osteomyelitis and soft tissue abscesses, [^11^C]methionine appeared to be more sensitive than [^18^F]FDG for identifying the lymph nodes draining the infected limb [6,7,8,9]. [^11^C]methionine accumulated in all enlarged lymph nodes, whereas [^18^F]FDG accumulated in 50% [8].

### 1.2. The Present Study

**Figure 1:**
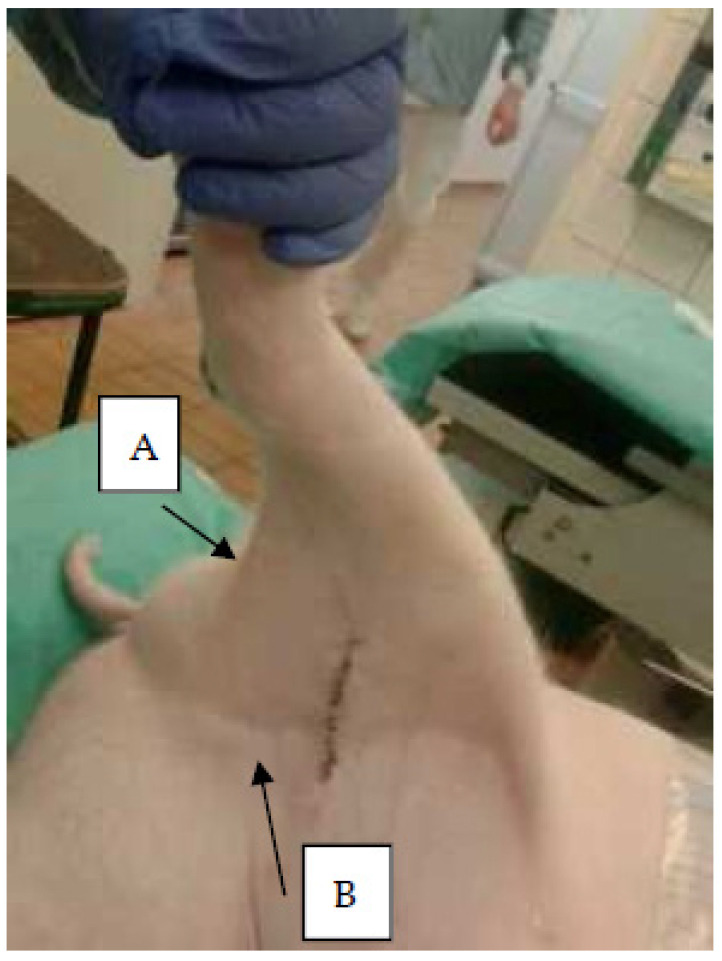
Lymph nodes. Photo of right hind limb of a juvenile pig. Arrows indicate positions of A, right popliteal; and B, right mammary lymph nodes. The medial iliac lymph nodes (right and left) are located within the abdominal cavity on the surface of the iliac bones (Figure 2).

**Figure 2:**
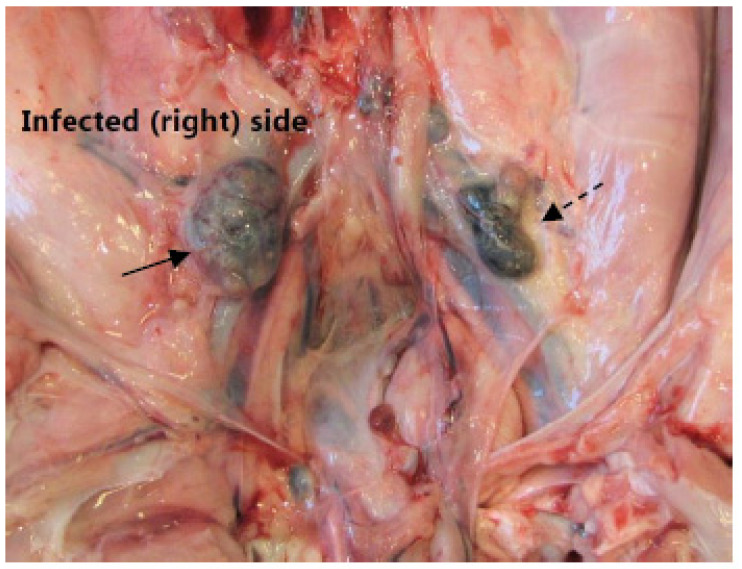
Medial iliac lymph nodes. A photo at necropsy of the caudal pig abdomen. The picture shows the right enlarged medial iliac lymph node (solid arrow) draining the infected hind limb. The lesser left lymph node (dashed arrow) drained the left non-infected left hind limb.

In the present study, we investigate and compare lymph nodes (Figure 1 and Figure 2) draining the infected and the contra-lateral non-infected hind limb in a juvenile porcine model of non-systemic *S. aureus* infection. This is done in vivo with PET/CT scans of tracer uptake, and ex vivo using lymph nodes from the animals after euthanasia and necropsy. The tracer uptake is measured as the maximum Standardized Uptake Value (SUV_max_) of [^18^F]FDG and [^11^C]methionine. After necropsy, we estimate area fractions of IHC-stained cells expressing the nuclear protein Ki-67 antigen, leukocyte L1 antigen complex (calprotectin), and interleukin-8 (IL-8) antigen.

The Ki-67 nuclear antigen marks proliferating cells expressed in all cycling cells except resting cells in the G0 phase. It thus reflects cells in the G2 and M phases, and to a lesser extent, the G1 and S phases of the cell cycle [10].

Inflammatory cells such as macrophages, monocytes, and neutrophils express the calcium-binding leukocyte calprotectin antigen L1 [11]. The L1 antigen has an innate defense function and possesses an antimicrobial effect on *S. aureus* and other microbes [11].

The neutrophilic granulocytes have high expression of two chemokine receptors, CXCR1 and CXCR2 [12], to which IL-8 binds with high affinity [13,14]. Lauersen et al. [15] have demonstrated by IHC on porcine samples that IL-8 is a predominantly neutrophil-related cytokine also present in epithelial and blood vessel endothelial cells of the inflammatory process.

Thus, the current study explores the influence of the infection on the draining lymph nodes by pairwise comparison of lymph nodes in the right (infected) and left (non-infected) sides. The lymph node weights; PET tracer uptake in the lymph nodes; and IHC analysis of Ki-67, L1, and IL-8 positive cells are compared.

## 2. Results

### 2.1. PET Scans

PET scanning with [^18^F]FDG was performed in all 20 pigs, but failed in 3 (Table 1). The failed scans represent cases where no or only faint [^18^F]FDG uptake, neither physiological nor pathological, was seen in the PET scan. We do not have any good explanations for that. Nine pigs (no. 1–9) also had a [^11^C]methionine PET/CT. For an overview, see Table 1.

**Figure 3:**
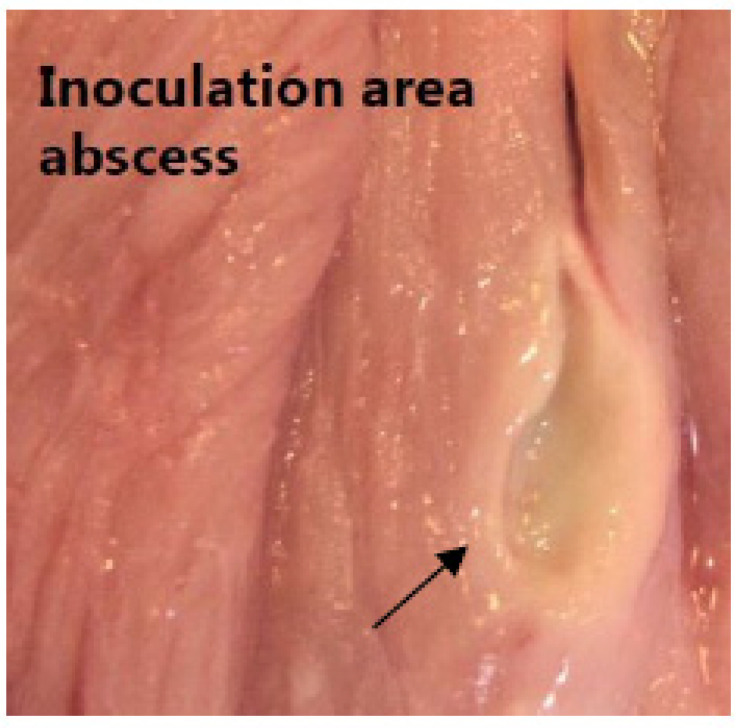
Inoculation abscess. A photo of a chronic lesion as identified by abscesses with peripheral capsular formation (arrow) at the inoculation site in between muscle tissue above the right femoral artery.

By gross pathology, all pigs developed osteomyelitis, 13/20 pigs developed periosseous abscesses, 11/20 developed inoculation site abscesses (Figure 3), and 6/20 developed arthritis in the right (inoculated) hind limb; only pig no. 14 had an infectious lesion in the left hind limb (subcutaneous abscess), and this lesion was not associated with the inoculation (Table 1).

### 2.2. Lymph Node Weights

The lymph nodes draining the hind limb are the superficial and deep popliteal lymph nodes, the subiliac lymph node, the superficial inguinal (mammary) lymph nodes, and the iliofemoral (medial iliac) lymph nodes. In our studies, osteomyelitis lesions were relatively easy to identify on CT and to find even small lesions and guide localization if missed on necropsy. For the lymph nodes, identification was more difficult. The medial iliac lymph nodes constitute the part that lies caudal to arteria circumflexa ilium profunda (Figure 2). In addition, the popliteal lymph nodes can be difficult to find, and otherwise, they are inconsistent (the superficial one is missing or a duplex, and the profound may be missing in half of the pigs). The most easily localizable lymph nodes were the mammary and subiliac lymph nodes. Tracer-accumulating lymph nodes were most often the popliteal and mammary lymph nodes; rarely the subiliac lymph nodes; and the medial iliac lymph nodes were not easy to identify in CT scans.

In Table 2, the lymph node weights are reported. In each of the subgroups of popliteal, mammary, and medial iliac lymph nodes, data were evaluated as right (infected side) versus left (non-infected control side), using non-parametric pairwise comparison (Wilcoxon). In all three groups, the median weight was highest on the right side, but for the popliteal lymph nodes, the difference was not statically significant.

### 2.3. FDG Uptake

The [^18^F]FDG uptake in the lymph nodes is reported as SUV_max_ values in Table 3. As for lymph node weights, there was a statistically significant difference between right and left (infected and non-infected) for the mammary and medial iliac lymph nodes, but not the popliteal lymph nodes.

### 2.4. FDG Uptake

For the nine pigs that were [^11^C]methionine-PET-scanned (Table 1), the SUV_max_ values are reported in Table 4. Only for the popliteal lymph nodes was the uptake significantly higher in the right than the left side.

### 2.5. Area Fractions

**Figure 4:**
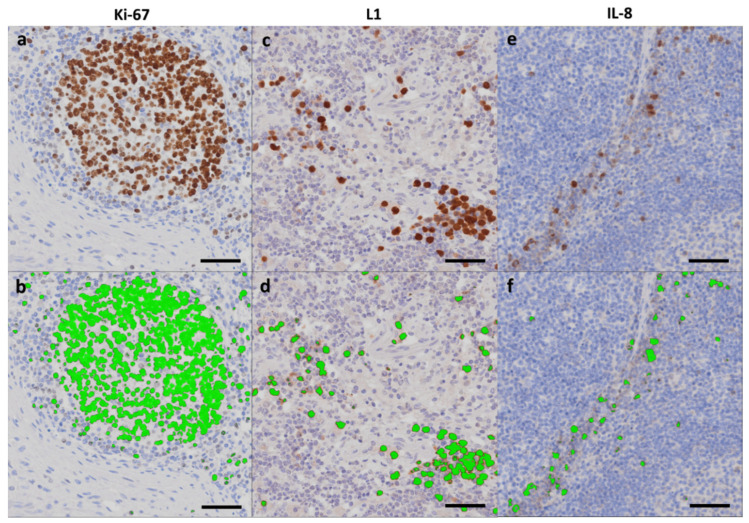
Area fractions. IHC stained lymph node tissue identifying Ki-67 (pig 3), L1 (pig 2), and IL-8 (pig 5) positive cells as indicated (**a**,**c**,**e**), and corresponding automatic threshold identification of positive cells with subsequent green labeling of the cells using Visiopharm Image Analysis Software (**b**,**d**,**f**). Scale bar = 50 µm.

The popliteal and mammary lymph nodes were Ki-67, L1, and IL-8 area fraction determined in the pigs, which were also [^11^C]methionine scanned. As an example of area fractions, see Figure 4. Area fractions for IHC stained cells from the lymph nodes are reported in Table 5.

In general, the median area fractions and volumes of activated tissues were higher in lymph nodes draining the infected hind limb compared with those draining the non-infected limb, but this was not always statistically significant. For the popliteal lymph nodes, the area fractions showed no clear pattern, in some cases being highest in the lymph node from the right side, in other cases from the left side. The differences were not statistically significant (detailed statistics not shown).

For the mammary lymph node pairs, the area fractions of Ki-67 and IL-8 were significantly higher in the right (infected) side compared to the left (non-infected control) side, whereas the differences on L1 were not statistically significant. For the volume of activated tissues, the difference was not significant for Ki-67, but was for L1 and IL-8.

### 2.6. Correlations

Correlations between area fractions (Ki-67, L1, or IL-8) and tracer uptake (SUV_max_) were not statistically significant for either [^18^F]FDG or [^11^C]methionine, neither for the right popliteal lymph nodes nor for the right mammary lymph nodes. Furthermore, the weights of these lymph nodes did not have statistically significant correlations with area fractions.

## 3. Discussion

### 3.1. Background Summary

Recently, we noticed that [^11^C]methionine, but not [^18^F]FDG, detected all enlarged lymph nodes in our juvenile porcine hematogenous *S. aureus* osteomyelitis model [7]. Accordingly, we wanted to examine and characterize the lymph nodes to explain this difference. Lymph nodes’ presence in pigs is variable or is difficult to identify [19]. The biological diversity or the difficulties have given rise to a lack of uniformity in sampling within pigs (right versus left side) and between pigs. Despite this, the results indicate that we can draw some conclusions.

### 3.2. Lymph Node Weights

We hypothesized that lymph nodes draining *S. aureus* infected foci, i.e., lymph nodes in the right (infected) hind limb would weigh more than in the non-infected (control) left hind limb (Figure 2). That could be a consequence of either cell proliferation (hyperplasia) or swelling caused by increased drainage in the infected tissue. The area fraction estimated in the present study is, to some extent, associated with the number of cells. However, in order to be able count the number of cells, a certain volume of tissue needs to be assessed. A volume of tissue within the microscope is obtained by cutting a very thick tissue-section, increasing the *z*-axis in the slide from the normal three micrometers to approximately twice the diameter of the cells to be counted. With the protocol used in the study here, counting the number of cells was not possible.

The lymph nodes were grouped as popliteal, mammary, or medial iliac (Figure 1, Figure 2). The median lymph node weight was higher on the infected (right) side than on the non-infected for all three groups, but the difference was only statistically significant for the mammary and medial iliac lymph nodes (Table 2).

As lymph vessels seem not to originate in the deep part of bone tissue (affected with osteomyelitis), but only from the superficial part and from soft connective tissue [19], the presence of a soft-tissue lesion in the right hind limbs in 13/20 pigs is probably the main reason for lymph node weight increase (Table 1 and Table 2).

In 11/20 pigs, an abscess had developed at the inoculation site (right proximal femoral artery), which may also have affected the draining lymph nodes (Table 1, Figure 3). The superficial popliteal lymph nodes, which drain the lower part of the limb, demonstrated no increase in weight in the right side (Table 2). The presence of primarily acute soft tissue lesions (phlegmons) in the distal hind limb was due to contiguous inoculation from osteomyelitis in contrast to, for example, the prevalent abscesses with distinct abscesses capsules at the inoculation site present in 11/20 pigs (drained by the mammary lymph node) (Table 1).

### 3.3. Accumulation of PET Tracers

We also hypothesized that lymph nodes in the infected leg would accumulate more [^11^C]methionine and [^18^F]FDG than those in the non-infected (control) limb.

By comparing the right (infected) side and the left (non-infected control) side, the right mammary and iliac lymph nodes had significantly higher [^18^F]FDG SUV_max_ than the left side nodes (Table 3). In the right–left pairs of superficial popliteal lymph nodes, however, there were no significant differences of [^18^F]FDG SUV_max_ in the nodes in the right and left hind limbs (Table 3). Among the nine [^11^C]methionine-PET scanned pigs, [^11^C]methionine SUV_max_ was significantly higher in the right than the left popliteal lymph nodes, but not so for the mammary and medial iliac lymph nodes (Table 4).

The difference in tracer uptake may be a result of the presence of primarily acute soft tissue lesions (phlegmons) in the distal hind limb due to contiguous inoculation from osteomyelitis, in contrast to, for example, the prevalent abscesses with distinct abscess capsules at the inoculation site present in 11/20 pigs (drained by the mammary lymph node), which may push the increase in size and [^18^F]FDG accumulation of the mammary lymph node (Table 1 and Table 3). The [^11^C]methionine accumulation in the popliteal lymph nodes may thus reflect drainage of the most acute lesions in contrast to the mammary node, which drained chronic lesions, as identified by abscesses with peripheral capsular formation (Figure 3, Table 4).

A bio-distribution study by Salber et al. investigating experimental bacterial infection in rats has shown increased uptake of [^3^H]methionine or [^11^C]methionine in infected tissue and the local draining lymph node compared to the non-infected tissue [20]. The histology of the infected tissue showed abscesses with necrosis and a band of neutrophils, macrophages, and remnants of muscle fibers. That is in line with Stöber et al., who showed that inflammatory cells take up methionine and more so after activation. Only 50% of the uptake into inflammatory cells was receptor-mediated, whereas for neoplastic cells, this was 90% [2].

Nakagawa et al. studied [^18^F]FDG accumulation in reactive neck lymph nodes in humans with oral cancer (false-positive nodes by staging), and demonstrated a positive correlation between SUV_max_ and the number of secondary follicles [21]. Further, these authors found by IHC that the follicular dendritic cells in the secondary follicles are probably responsible for [^18^F]FDG accumulation, as these cells are positive for glucose transporter type 1. Thus, the accumulation of [^18^F]FDG in mammary lymph nodes in pigs draining the infected limb is probably also due to the presence of an increased number of secondary follicles. This further seems in agreement with our finding of an increase in proliferative response (Ki-67 IHC) in a sub-fraction of the mammary nodes, and that the more chronic lesions drained to the mammary lymph nodes. However, we did not find a correlation between Ki-67 area fractions and [^18^F]FDG accumulation (Table 5).

Most amino acids exist in L and D forms, but animal and human physiology use only the L-form [21]. Most radiotracer studies on methionine, including the present study, use L-methionine. We found higher uptake of enlarged popliteal lymph nodes (*n* = 9, Table 4) and a fair uptake in osteomyelitis lesions compared to FDG [8]. However, Friedman mentions that D-amino acids are generally part of bacterial cell walls, contributing to their resistance to digestion by the body’s (L-form) proteolytic enzymes [22]. Both *E. coli* and *S. aureus* have a high uptake of D-methionine, whereas the D-form has low uptake in animal and human tissue, for which reason, D-[^11^C]methionine in a mouse model showed much more infection-specific uptake than L-[^11^C]methionine [23]. The group has since then developed a new synthesis for D-[^11^C]methionine, performed in vitro tests showing uptake of the tracer in a broad range of clinically relevant bacteria, and are preparing for in-human evaluation of D-[^11^C]methionine in patients with vertebral infections [24]. It will be interesting to see the potential of D-form amino acids as infection tracers in *S. aureus* infections.

### 3.4. Area Fractions

The study showed that area fractions of Ki-67 and IL-8 positive cells were significantly increased in mammary lymph nodes draining infected limbs (Table 5). The increase in area-fraction of Ki-67 or IL-8 may be an effect of an increase in the number of cells (hyperplasia) or an increase in cell size (hypertrophy). However, no statistically significant correlation was found between the accumulation of either of the two tracers ([^11^C]methionine and [^18^F]FDG) and area fractions of Ki-67, L1, or IL-8 positive cells (Table 5).

In inflammatory and infectious lesions in pigs, IL-8 is present in neutrophils, epithelial cells of the respiratory system, and endothelial cells of newly formed blood vessels in granulation tissue [25]. Thus, the increase in area fractions of IL-8 positive cells of mammary lymph nodes is probably an effect of the activation and migration of neutrophils. We have also previously reported that IL-8 plays a role in OM, as [99mTc]Tc-IL-8 detects 70% of OM lesions compared to a 100% sensitivity of [^18^F]FDG PET/CT [17].

As there were no correlations between the weights of popliteal lymph nodes or mammary lymph nodes and area fractions of Ki-67, the increase in weight may represent hypertrophy of the cells of the nodes, or more likely, increased drainage to the lymph node resulting in dilated lymphatic vessels and sinuses (Table 2).

The lack of an increase of area fractions in the superficial popliteal lymph nodes, which contrasts the findings in the mammary lymph nodes, may also relate to the small size of the popliteal nodes, as small nodes will result in the generation of only a few slabs of tissue for staining, which could result in inhomogeneous sampling.

### 3.5. Limitations

The study has several limitations.

Translation of our results to human lymph nodes may not be directly possible, as the histological structure of pig lymph nodes is different from, for example, human lymph nodes: it is the so-called inverted lymph node that is also present in elephants, dolphins, hippopotamus, and warthogs [26]. In the pig, the medullar tissue is in the periphery of the lymph node, and the cortical part is in the central area. Therefore, T- and B-lymphocytes are found mainly in a translational atypical localization. The cortex, paracortex, and medulla are distinct micro-anatomical structures [25]. Neonatal germ-free piglets do not have germinal centers. A comparison of the lymph node structures in different species has been summarized elsewhere [27,28]. A positive aspect of pig lymph nodes is that one lymph node drains to the next in line: superficial inguinal to deep inguinal, and this is similar to the structure in humans, but not typical in mice. Another difference is the large central cisternae and intra-trabecular lymph channels with lymphatic valves in pig lymph nodes [29]. These are unique findings not known from the lymph nodes in other species. However, we do not know if these differences are essential for our study.

The experiment was performed exclusively on female juvenile pigs in order to place urinary catheters during prolonged anesthesia, as that is not possible in male pigs.

We would have preferred to examine the more robust parameter SUV_peak_ rather than SUV_max_ [30,31], but the parameter was not available in the old software used. It may also have been more relevant to investigate pathological [^18^F]FDG and [^11^C]methionine accumulation instead of absolute SUV_max_ values. There is general agreement on FDG cut-offs of SUV_max_ values of 2.5. There is, however, no consensus on cut-off values of methionine.

The IHC results may depend on the duration of fixation that varied both intra- (different tissues) and inter-pigs. However, the fixation protocol (including the time that tissues were in the fixative) was similar for the lymph nodes from the right and left limbs of the same pig. That makes comparisons between the contra-lateral sides reasonable. Variation in shrinkage percentage may rely on other factors, e.g., the “activity” of the lymph node, which could have influenced the area fractions of IHC positive cells, obscuring minor differences between the left and right sides.

We would have preferred to do quantitative IHC in all cases. However, the procedure was cumbersome. We, therefore, only managed to do quantitative IHC in one third of the cases. We would have preferred to do estimates of the number of antigen positive cells, rather than area fractions. We would also have preferred to have [^11^C]methionine-PET scanned more pigs.

Future studies should include more [^11^C]Methionine-scanned pigs, but the methods described here to find evidence for the identity of cells or cell function responsible for tracer accumulations seem to work, although by an indirect method.

## 4. Materials and Methods

### 4.1. Pigs and Porcine Model

The present study reports data from a multi-tracer OM study in juvenile pigs [6]. The present study includes 20 pigs from previous papers on the multi-tracer study. The propofol-anesthetized pigs were inoculated with the porcine strain S54F9 of *S. aureus* (8000–30,000 CFU/kg) into the right femoral artery in the groin region to induce hematogenous regional osteomyelitis in the right hind limb only [32,33,34]. The pigs were treated with painkillers. Humane endpoints for immediate euthanasia were: clinical signs of systemic disease (fever for more than 24 h, shallow respiration), anorexia or reluctance to drink for more than 24 h, more than 10% body weight loss, pain untreatable with an opioid analgesic, or refusal to stand.

After 6 to 14 days, the pigs were propofol-anesthetized and PET/CT-scanned, followed by euthanasia and necropsy.

### 4.2. Preparation of Tracers

The ^11^C and ^18^F radionuclides were produced at the Department of Nuclear Medicine and PET in Aarhus using either a PETtrace 800 series cyclotron (GE Healthcare, Uppsala, Sweden) or a Cyclone 18/18 cyclotron (IBA, Louvain-La-Neuve, Belgium). [^18^F]FDG was produced by a standard procedure applying a GE Healthcare MX Tracerlab synthesizer, Mx cassettes supplied by Rotem Industries (Arava, Israel), and chemical kits provided by ABX GmbH (Radeberg, Germany). [^11^C]methionine was synthesized as previously described [35].

### 4.3. PET and CT

Pigs in dorsal recumbency had an initial scout view to secure body coverage from snout to tail. [^18^F]FDG PET/CT was performed at either the Department of Nuclear Medicine and PET in Aarhus or the Department of Nuclear Medicine in Aalborg. All [^11^C]methionine PET/CTs took place in Aarhus.

In Aarhus, all examinations were in a Siemens Biograph TruePoint™ 64 PET/CT scanner (Siemens Healthineers, Erlangen, Germany). Reconstruction was with four iterations, 21 subsets, and a 3-mm Gaussian post-processing filter. The voxel size was 2 × 2 × 2 mm^3^ in a 336 × 336 matrix.

In Aalborg, two different scanners were used because of a scanner replacement during the project. The first 15 pigs were scanned on a GE VCT Discovery True 64 PET/CT scanner (GE Healthcare, Chicago, Illinois, USA). Reconstruction used 2 iterations, 28 subsets, and a 6 mm Gaussian filter. The voxel size was 5.5 × 5.5 × 3.3 mm^3^ in a 128 × 128 matrix. The last five pigs (no. 16–20 in Table 1) were scanned on a Siemens Biograph mCT (Siemens, Erlangen, Germany) with time-of-flight (TOF) detection. The reconstruction parameters were 3 iterations, 21 subsets, and a 3-mm Gaussian filter. The voxel size was 1.02 × 1.02 × 2.03 mm^3^ in a 400 × 400 matrix.

### 4.4. Reading the Scans

[^18^F]FDG and [^11^C]methionine PET scans were read individually and as fused images with CT. In the mammary, medial iliac, and popliteal lymph nodes, tracer-accumulation was the maximal Standardized Uptake Value (SUV_max_) noted.

### 4.5. Gross Pathology and Diagnostics of Lesions

Table 1 presents details on pathology and microbiology. In all pigs, the superficial popliteal, mammary, and medial iliac lymph nodes (right and left) were excised, trimmed, weighed, and fixed in 3.7% neutral buffered formaldehyde for a minimum of four days.

### 4.6. Immunohistochemistry

In an attempt to measure indicators of cell activity quantitatively, we estimated area fractions of Ki-67, L1, and IL-8 antigens in popliteal and mammary lymph nodes from the pigs that were PET-scanned with both [^11^C]methionine and [^18^F]FDG radionuclides. Thus, superficial popliteal and mammary lymph nodes were prepared for IHC analysis. Starting from an arbitrary direction, each lymph node was systematically divided (systematic-random cutting) into 3–4 mm thick slabs, which, depending on the size of the lymph node, gave rise to a total of 2–14 slabs from each lymph node. Slabs were placed in tissue cassettes and then dehydrated and paraffin-wax-embedded. Five consecutive tissue sections (3–5 μm thick, three sections of interest and two negative controls) were cut from all slabs and mounted on adhesive glass slides (Superfrost Plus, Thermo Scientific, Gerhard Menzel B.V. & Co. KG, Braunschweig, Germany).

IHC for detection of Ki-67 antigen (cell proliferation) was according to instructions given by the antibody producer, except for increasing the antigen retrieval time based on optimization experiments. IHC was modified from previously published protocols for detection of L1 antigen (macrophages, monocytes, and neutrophils) and IL-8 antigen (activation of macrophages and neutrophils) [36,37].

Briefly, all lymph node sections were deparaffinized, rinsed with distilled water, and, between all steps of the subsequent staining procedure, rinsed with Tris-buffered saline (TBS), pH 7.6. For Ki-67 staining, tissue sections were pretreated in Tris-EGTA buffer pH 8.0 for 4 × 5 min by microwave oven heating, and then kept for 15 min in the Tris-EGTA buffer. Sections were incubated overnight at 4 °C with the primary anti-Ki-67 antibody (clone MIB-1, M7240, DakoCytomation, Glostrup, Denmark) diluted 1:200 in 1% Bovine serum albumin (BSA)/TBS.

For L1 staining, tissue sections were pretreated with Tris-EDTA buffer pH 9.0 for 2 × 5 min by microwave oven heating, and then rested for 15 min with the Tris-EDTA buffer. Tissue sections were incubated overnight at 4 °C with primary anti-L1 antibody (clone MAC387, MCA874G, Bio-Rad Laboratories, Irvine, CA, USA) diluted 1:500 in 2% BSA/TBS.

For IL-8 staining, sections were pretreated by pressure cooking using DIVA decloaking buffer (DV2005L2J, Biocare Medical, Concord, CA, USA). Tissue sections were incubated overnight at 4 °C with primary anti-IL-8 antibody (Clone 8M6, MCA1660, Bio-Rad Laboratories, Irvine, CA, USA) diluted 1:1000 with 1% BSA/TBS.

After the pretreatments (antigen retrieval), as described for each staining protocol, all tissue sections were blocked for endogen peroxidase in 0.6% H_2_O_2_ for 15 min, and in UltraVision Block (TL-125-HL, Thermo Fisher Scientific, Waltham, MI, USA) for five minutes. The primary antibody binding was detected using the UltraVision detection kit (TL-125-HL, Thermo Fisher Scientific, Waltham, MI, USA) with the 3-amino-9-ethyl carbazole (AEC) chromogen for Ki-67 and IL-8, and the 3,3′-diaminobenzidine (DAB) chromogen for L1. Mayer’s Hematoxylin counterstaining followed for ten seconds, rinsing with tap water for one minute, rinsing with distilled water for four minutes, and then mounting with glycerol-gelatin.

Positive control tissue included tissue sections from formalin-fixed normal pig intestine (Ki-67 staining of proliferating enterocytes) and lymph node (L1 staining), and sections from formalin-fixed porcine lung tissue with abscesses (cells expressing IL-8). Negative controls were performed on parallel tissue sections, replacing the primary antibodies with a nonsense antibody of a similar isotype, IgG1 (X0931, Agilent/Dako, Santa Clara, CA, USA) for Ki-67 and L-1, and IgG2a (X0943, Agilent/Dako, Santa Clara, CA, USA) for IL-8. Nonsense antibodies at the same concentration as the primary ones were applied.

An estimate of the amount of activated lymph node tissue, as indicated by Ki-67, L1, and IL-8, was also calculated by multiplication of area fractions by lymph node weight.

### 4.7. IHC Image Analysis

The slides were then IHC-captured as digital images using a combined Olympus microscope and camera (Olympus BX51, XC10, U-CMAD3, Tokyo, Japan). Area fractions of IHC positive cells relative to the total tissue area were estimated in Visiopharm Image Analysis Software (Visiopharm, Hoersholm, Denmark). Briefly, regions of interest (ROIs) were drawn manually along the lymph node capsule or in deeper parts excluding missing tissue, followed by the application of a predefined filter for enhancement of the AEC- or DAB-stained cells, automatic threshold identification of the dark brown color (IHC positive cells), and subsequent green labeling of these cells (Figure 4) [38]. Then, stained areas, as well as reference areas in all ROIs in all slabs, were added. Finally, we calculated the individual lymph node area fraction.

### 4.8. Statistical Analysis

Non-normal distribution, non-parametric tests (Wilcoxon’s signed rank test instead of normal paired *t*-test, Spearman’s correlation coefficient instead of normal Pearson).

## 5. Conclusions

We found increased lymph node weights, accumulation of [^18^F]FDG and [^11^C]methionine, and increased area fractions of Ki-67 and IL-8, but not L1, in lymph nodes draining soft tissues infected with *S. aureus*. The increased weights, accumulation of [^18^F]FDG, and increased Ki-67 area fractions could relate to a hypertrophic (as lymph node weight and Ki-67 did not correlate) or, perhaps more likely, increased drainage to the lymph node resulting in dilated lymphatic vessels and sinuses. The increase in [^11^C]methionine may indicate a more acute lymph node response, although the cells responsible remain elusive. Future studies attempting to identify inflammatory cells capable of accumulating the PET tracers, especially [^11^C]methionine, using the methods described here may benefit from increasing the number of methionine scans and IHC-examined lymph nodes, and using inoculation of bacteria and maybe dye in soft tissue draining to the easily identifiable mammary lymph node.

## Figures and Tables

**Table 1:** PET/CT scans and lesions by pathology.

Pig ^1^	Body Weight (kg)	PET Tracer Applied ^2^	Days from Inoculation to PET Scan	Right Hind Limb				Left Hind Limb, Gross Lesions
				Osteomyelitis Sites (*n*) ^3^	Soft Tissue Lesions			
					Peri-Osseous Abscesses (*n*)	Abscess at Inoculation Site (+/-)	Arthritis Sites (*n*)	
1	40	FDG, Met	7	4	1	+	0	-
2	40	FDG, Met	7	2	1	-	1	-
3	42	FDG, Met	7	1	1	+	1	-
4	20	FDG, Met	14	1	0	-	0	-
5	22	FDG, Met	7	2 ^4^	0	-	0	-
6	23	Met	7	5	0	-	0	-
7	21	Met	8	5	1	-	0	-
8	23	FDG, Met	8	4	1	-	0	-
9	22.5	FDG, Met	7	5	2	-	1	-
10	21	FDG	7	3	4	+	0	Fibroma at calcaneus
11	18.3	FDG	7	4	3	+	2	-
12	22.0	FDG	6	3	1	+	0	-
13	22.1	FDG	6	2	1	+	0	-
14	23.6	FDG	8	4	0	-	0	Subcutaneous abscess at calcaneus ^5^
15	23.6	FDG	8	3	0	-	0	-
16	19.3	FDG	8	3	0	+	0	-
17	20.1	-	8	5	0	+	3	-
18	19.0	FDG	9	1	1	+	2	-
19	19.0	FDG	9	2	2	+	0	-
20	20.5	FDG	8	3	2	+	0	-

^1^ Pigs are numbered consecutively. Previous publications have reported data on osteomyelitis, including microbiological reisolation of *S. aureus*, on pigs 1–3 ([6,7]), pigs 5–9 ([8]), pigs 1–8 ([9,16]), pig 18–20 ([17]), and pigs 1–3 and 5–20 ([18]). ^2^ FDG is [^18^F]FDG, and Met is [^11^C]methionine. ^3^ The sectioning technique used to identify osteomyelitis generally applied one mid-sagittal cut in each of the bones constituting the hind limbs. ^4^ One additional osteomyelitis lesion was identified in the proximal part of the right humerus. ^5^ *S. aureus* found by cultivation, but not the strain inoculated. +: presence. -: absence.

**Table 2:** Lymph node weights.

Pig ID	Popliteal Weight (g)		Mammary Weight (g)		Medial Iliac Weight (g)	
	Right	Left	Right	Left	Right	Left
1	0.94	0.65	-	-	3.25	1.48
2	0.77	0.68	-	-	5.72	1.67
3	0.45	0.95	-	-	4.82	2.49
4	-	-	4.32	3.33	2.62	2.31
5	-	-	3.13	4.37	2.61	2.87
6	0.63	0.32	4.48	3.44	0.63	0.32
7	1.79	0.99	3.85	2.60	4.41	1.67
8	1.36	-	3.45	2.25	1.00	0.76
9	1.10	0.05	3.98	1.17	1.18	0.47
10	1.63	1.13	3.86	3.33	2.57	1.00
11	0.53	0.13	4.48	2.11	1.09	0.92
12	1.57	0.56	6.62	2.46	2.01	1.69
13	0.70	0.56	2.63	2.62	1.59	1.88
14	0.40	1.13	4.00	4.28	-	-
15	0.30	0.37	2.30	1.88	-	-
16	0.11	-	2.13	1.85	-	-
17	-	-	4.52	1.75	-	-
18	-	-	3.51	3.83	-	-
19	-	-	4.23	3.51	-	-
20	-	-	-	-	-	-
**Median**	0.735	0.605	3.92	2.61	2.57	1.67
**R vs. L**	*p* = 0.092 (NS)		*p* = 0.009		*p* = 0.007	

R = Right (infected side), L = Left (non-infected control side), NS = not significant.

**Table 3:** Maximum Standardized Uptake Values (SUV_max_) of [^18^F]FDG in lymph nodes.

Pig ID	Popliteal SUV_max_		Mammary SUV_max_		Medial Iliac SUV_max_	
	Right	Left	Right	Left	Right	Left
1	0.8	0.4	1.1	0.7	1.1	0.9
2	0.4	0.3	7.4	4.9	1.6	1.5
3	1.0	1.0	0.9	0.8	3.8	1.0
4	-	-	1.2	0.9	1.2	0.9
5	0.6	0.7	2.6	1.3	2.6	1.4
6	-	-	-	-	-	-
7	-	-	-	-	-	-
8	3.8	0.3	0.9	0.8	1.0	0.8
9	1.5	0.6	3.0	1.0	2.2	2.0
10	5.5	3.5	2.6	3.1	3.6	1.6
11	1.2	0.5	2.2	2.1	1.5	1.6
12	1.8	0.7	3.6	1.1	-	-
13	0.8	0.5	1.1	0.8	1.2	1.3
14	1.0	2.0	1.4	1.5	-	-
15	0.6	0.7	1.9	0.7	-	-
16	0.9	0.4	1.4	1.5	-	-
17	-	-	-	-	-	-
18	0.9	1.4	1.9	1.2	-	-
19	0.6	0.7	3.2	2.4	-	-
20	0.6	1.4	3.5	2.4	-	-
**Median**	0.9	0.7	1.9	1.2	1.55	1.35
**R vs. L**	*p* = 0.19 (NS)		*p* = 0.003		*p* = 0.016	

R = Right (infected side), L= Left (non-infected control side), NS = not significant.

**Table 4:** Maximum Standardized Uptake Values (SUV_max_) of [^11^C]methionine in lymph nodes.

Pig ID	Popliteal SUV_max_		Mammary SUV_max_		Medial Iliac SUV_max_	
	Right	Left	Right	Left	Right	Left
1	2.1	1.1	4.6	2.6	4.6	2.6
2	7.5	3.4	1.8	2.8	3.3	4.0
3	1.2	1.0	1.1	1.1	7.5	4.1
4	-	-	-	-	0.1	0.1
5	0.9	1.1	1.4	1.3	1.3	2.4
6	1.4	0.4	1.3	2.4	1.4	0.4
7	3.5	1.3	1.6	0.9	1.9	0.6
8	3.9	0.3	1.7	1.0	1.7	1.1
9	2.9	0.6	3.4	0.5	3.4	0.5
**Median**	2.5	1.05	1.65	1.2	1.9	1.1
**R vs. L**	*p* = 0.021		*p* = 0.33 (NS)		*p* = 0.093 (NS)	

R = Right (infected side), L = Left (non-infected control side), NS = not significant.

**Table 5:** Area fractions of the mammary and the popliteal lymph nodes.

Pig ID	Lymph Node	Area Fraction (%)					
		Ki-67		L1		IL-8	
		Right	Left	Right	Left	Right	Left
1	popliteal	8.38	4.88	6.36	2.19	0.20	0.19
2	popliteal	6.43	5.69	4.20	1.53	0.18	0.45
3	popliteal	5.17	7.91	0.37	0.19	0.09	0.12
6	popliteal	30.61	6.91	0.11	0.11	2.10	0.14
7	popliteal	3.03	2.52	0.37	0.58	0.29	0.02
8	popliteal	7.40	-	1.07	-	0.21	-
9	popliteal	5.23	-	2.76	-	0.26	-
**Median**		6.43	5.69	1.07	0.58	0.21	0.14
**R vs. L**		(NS)		(NS)		(NS)	
4	mammary	13.21	9.18	0.25	0.33	0.10	0.08
5	mammary	13.18	12.34	6.44	1.06	0.56	0.21
6	mammary	12.01	5.83	0.53	0.40	0.29	0.28
7	mammary	2.89	1.45	0.77	0.62	0.37	0.36
8	mammary	1.97	1.79	0.37	0.51	0.06	0.06
9	mammary	3.28	2.03	2.52	3.42	0.25	0.09
**Median**		7.6	3.9	0.65	0.57	0.27	0.15
**R vs. L**		* p * = 0.028		* p * = 0.75 (NS)		* p * = 0.028	

R = Right (infected side), L = Left (non-infected control), NS = not significant.

## Data Availability

Data can be supplied upon request.

## Abbreviations

AEC	Amino-ethyl-carbazole
CT	Computed tomography
DAB	3,3-Diaminobenzidine
FDG	2-flouro-2-deoxyglucose
IHC	Immunohistochemistry
Met	Methionine
OM	Osteomyelitis
PET	Positron emission tomography
ROI	Region of interest
*S. aureus*	*Staphylococcus aureus*
SPECT	Single photon emission computed tomography
SUV	Standardized uptake value
